# CD40 Signaling Promotes CXCR5 Expression in B Cells via Noncanonical NF-*κ*B Pathway Activation

**DOI:** 10.1155/2020/1859260

**Published:** 2020-07-31

**Authors:** Chuan Wei, Ying Chen, Lei Xu, Beibei Yu, Di Lu, Yanxiong Yu, Zhigang Lei, Rui Tang, Sha Zhou, Jifeng Zhu, Xiaojun Chen, Chuan Su

**Affiliations:** State Key Lab of Reproductive Medicine, Jiangsu Key Laboratory of Pathogen Biology, Department of Pathogen Biology and Immunology, Center for Global Health, Nanjing Medical University, Nanjing, Jiangsu 211166, China

## Abstract

Chemokine receptor CXCR5-mediated control of B cell trafficking in the lymphoid tissues plays a central role in orchestrating the B cell function, which not only guides the colocalization of B cells with follicular helper T cells in the follicular mantle zone but also determines the position of germinal center dark and light zones. However, the mechanisms that regulate the expression of CXCR5 in B cells remain unclear. Here, we show that the expression level of CXCR5 in B cells was substantially reduced in vitro culture conditions, while being maintained in the presence of CD40 signals. Furthermore, CD40 signaling promotes CXCR5 expression in B cells at least partially through noncanonical NF-*κ*B signaling pathway activation. However, other non-B cells also contributed to the optimal expression of CXCR5 in B cells through cell-cell contact and cytokine secretion. Our findings suggest that CD40 signaling-mediated activation of the noncanonical NF-*κ*B pathway promotes the expression of CXCR5 in a B cell-intrinsic way to orchestrate the trafficking of B cells.

## 1. Introduction

B cells are key players of the adaptive humoral immune, with roles including supporting follicular helper T-cell generation and producing antibodies [1, 2]. The outcome of these functions relies on the finely tuned traffic of B cells [3]. CXC chemokine receptor 5-mediated signals play a central role in orchestrating B cell trafficking in the lymphoid tissues, such as spleen and lymph nodes [4]. Indeed, CXCR5 signaling directly guides the colocalization of B cells with CXCR5^+^ T cells in the follicular mantle zone to support the generation of follicular helper T cells [5]. In addition, CXCR5 signaling directs B cells to the light zone and determines the position of germinal center dark and light zones, which segregate cells undergoing somatic hypermutation and antigen-driven selection to guide the germinal center reaction [6, 7]. Although CXCR5-mediated control of B cell trafficking plays a cardinal role in orchestrating the B cell functions, little is known about the underlying mechanisms that regulate the expression of CXCR5 in B cells.

CD40-mediated signals in B cells are particularly important for germinal center formation and humoral immune responses by orchestrating B cell functions [8]. Indeed, CD40 signaling plays multiple roles in B cell activation, proliferation, class-switch recombination, and affinity maturation [9]. The germinal center reaction is severally impaired in the absence of CD40-mediated signals and individuals with defective CD40-mediated signals are suffering from hyper-IgM syndrome with reduced germinal centers and class-switched B cells [10], which support the importance of CD40 signals in the generation of germinal center and high-affinity antibody responses. Given that CD40 is essential for CXCR5 expression in MDSCs [11], we wonder whether CD40 signaling is also involved in the regulation of CXCR5 expression in B cells.

In this study, we found a remarkably reduced expression of CXCR5 in B cells in vitro culture conditions, while being maintained in the presence of CD40 signal. Indeed, CD40 signaling promotes the expression of CXCR5 in a B cell-intrinsic way in part via noncanonical NF-*κ*B pathway activation. In addition, our results showed that other non-B-cell splenocytes were also involved in the optimal expression of CXCR5 in B cells through cell-cell contact and cytokine secretion. Our findings suggest that CD40-mediated expression of CXCR5 may contribute to the regulation of B cell trafficking.

## 2. Materials and Methods

### 2.1. Mice

Male wild-type C57BL/6 mice were obtained from the Animal Core Facility of Nanjing Medical University (Nanjing, China) and housed under specific pathogen-free conditions in accredited animal facilities at Nanjing Medical University. All procedures were conducted in accordance with the Regulations for the Administration of Affairs Concerning Experimental Animals (1988.11.1). All animal procedures were approved by the Institutional Animal Care and Use Committee (IACUC) of Nanjing Medical University for the use of laboratory animals (Permit Number: IACUC-1701019).

### 2.2. Flow Cytometry

Cells were isolated from spleens using mechanical disruption followed by red blood cell (RBC) lysis. Fc receptors of cells were blocked with anti-mouse CD16/CD32 (eBioscience, San Diego, CA) for 15 minutes at 4°C prior to antibody staining. Then, cells were incubated for 30 min at 4°C with the following antibodies: CD19-FITC (eBioscience), CXCR5-APC (BD Biosciences, San Jose, CA), and CD40-PE (eBioscience). After washing twice with PBS containing 1% FBS, cells were characterized using a FACSVerse cytometer (BD Biosciences). Data were analysed with FlowJo (Tree Star, version 10.0.7).

### 2.3. *In Vitro* Cell Coculture and Stimulation

B cells were purified from splenocytes using anti-B220 conjugated magnetic beads (Miltenyi Biotec GmbH, Bergisch Gladhach, Germany). Unless specifically noted otherwise, the purified B cells or splenocytes were stimulated *in vitro* by agonistic anti-mouse CD40 antibody (500 ng/mL; BioLegend, San Diego, CA), Recombinant BAFF (200 ng/mL; Thermo Fisher Scientific, Waltham, MA), or NIK inhibitor (10 ug/mL; MedChemExpress, Shanghai, China) for 72 hours. In parallel cultures, B cells were separated from non-B cell splenocytes by a porous (0.4 mm) membrane in otherwise identical conditions.

### 2.4. Western Blots

Splenocytes treated with anti-CD40 in the presence or absence of NIK inhibitor for 72 hours were homogenized in lysis buffer (Cell Signaling Technology, Danvers, MA) with 1 mM PMSF (Beyotime Biotech, Nantong, China). The protein concentration was determined using a bicinchoninic acid (BCA) Protein Assay kit (Thermo Fisher Scientific). Immunoblotting was performed using the following antibodies: anti-GAPDH (Abcam, Cambridge, MA), anti-p100/p52 (Cell Signaling Technology), and anti-RelB (Santa Cruz Biotechnology, Santa Cruz, CA).

### 2.5. Statistical Analysis

Statistics and graphing were conducted in GraphPad Prism 5.0 software. Error bars indicate s.d. Data were analyzed by two-tailed unpaired Student's *t*-test for two-group comparison and ANOVA test for three or more-group comparison. *P* value of less than 0.05 was considered significant.

## 3. Results and Discussion

### 3.1. CXCR5 Was Gradually Reduced in B Cells *In Vitro* Culture Conditions

**Figure 1:**
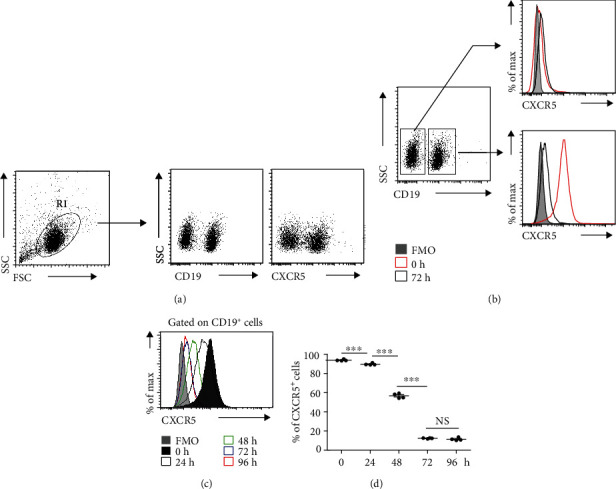
CXCR5 expression was reduced in B cells *in vitro* culture. Splenocytes were *in vitro* cultured for 24, 48, 72, and 96 hours. (a) Cells were stained with CD19-FITC and CXCR5-APC; (b) Representative flow cytometry data plots showed the levels of CXCR5 on CD19^+^ cells or CD19^−^ cells (representing three independent experiments, *n* = 4); (c) Representative flow cytometry data plots showed the mean fluorescence intensity (MFI) of CXCR5 on CD19^+^ cells *in vitro* cultured for 24, 48, 72, and 96 hours (representing three independent experiments, *n* = 4); (d) Flow cytometry data statistics showed the frequencies of CXCR5^+^ B cells (representing three independent experiments, *n* = 4). Living cells were gated according to forward scatter (FSC) and side scatter (SSC) parameters. All flow cytometry results were analysed and plotted using Fluorescence Minus One controls (FMO). ^∗∗∗^*P* < 0.001, NS indicating not significant (ANOVA test).

To study the underlying mechanisms that regulate the expression of CXCR5 in B cells, splenocytes were isolated from normal mice and cultured *in vitro*. The levels of CXCR5 in B cells were detected with flow cytometer by staining CD19 and CXCR5 (Figure 1(a)) and displayed a dramatic reduction in a time-dependent manner, while freshly isolated B cells displayed constitutive CXCR5 expression, reflected by mean fluorescence intensity (MFI) of CXCR5 (Figures 1(b) and 1(c)) and frequencies of CXCR5^+^ cells (Figure 1(d)) in B cells, suggesting that the microenvironmental cues *in vivo* were necessary for maintaining CXCR5 expression in B cells.

### 3.2. CD40 Signaling Promoted CXCR5 Expression in a B Cell-Intrinsic Way

Given that B cells are constantly exposed to BAFF stimulation *in vivo* [12], we first wondered whether BAFF had an ability to maintain CXCR5 expression in B cells. The results showed that the expressions of CXCR5 in B cells in the presence of BAFF were maintained slightly better than those in the absence of BAFF (Supporting Information Figure 1). These results suggested that BAFF may be not a major contributor to maintain the high expression levels of CXCR5 in B cells. The other factors for maintaining CXCR5 in B cells needed to be further investigated.

**Figure 2:**
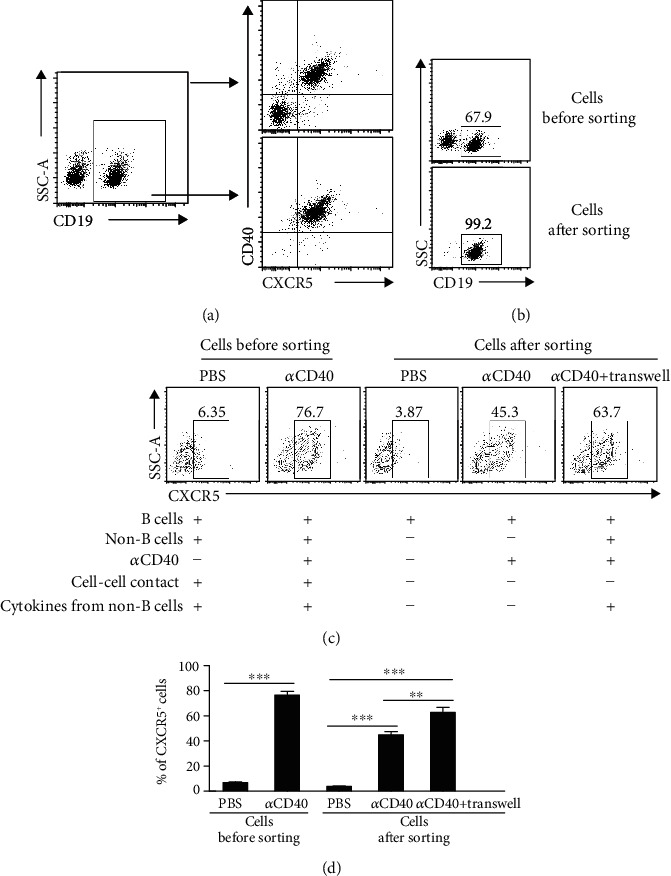
CD40-mediated CXCR5 expression was B cell intrinsic. (a) Representative flow cytometry data plots showed the coexpression of CXCR5 and CD40 on freshly isolated splenocytes or CD19^+^ cells (representing three independent experiments, *n* = 3); (b) The purity of B cells sorted by the magnetic cell sorting was measured; (c, d) The purified B cells or total splenocytes were stimulated with anti-mouse CD40 antibody for 72 hours. In the transwell system, CD19^−^ splenocytes from normal mice were cultured in the upper chambers, while CD19^+^ cells were cultured in the lower chambers. (c) Representative flow cytometry data plots represent the frequencies of CXCR5^+^ cells within the CD19^+^ cell population (representing three independent experiments, *n* = 3); (d) Data are representative of three independent experiments, *n* = 3; Living cells were gated according to FSC and SSC parameters. All flow cytometry results were analysed and plotted using FMO. ^∗∗∗^*P* < 0.001 (ANOVA test).

Since CD40 signaling has multiple roles in orchestrating B cell functions, we wondered whether CD40 signaling might contribute to the induction of CXCR5 expression in B cells. To test this hypothesis, we analyzed the expressions of CD40 and CXCR5 in B cells and found a coexpression of CD40 and CXCR5 (Figure 2(a)), suggesting that CD40 signaling might be involved in the regulation of CXCR5 expression in B cells. Moreover, we cultured splenocytes in the presence of CD40 signals and found that CD40 signaling promoted CXCR5 expression in B cells *in vitro*, reflected by frequencies of CXCR5^+^ cells (Supporting Information Figures 2(a) and 2(b)) and MFI of CXCR5 (Supporting Information Figures 2(c) and 2(d)) in B cells. To evaluate whether CD40 signaling directly affected the expression of CXCR5 in B cells, B cells were sorted by the magnetic cell sorting. The purity of sorted B cells was determined to be greater than 99% (Figure 2(b)). Of note, CD40 signaling promoted the expression of CXCR5 by directly targeting B cells (Figures 2(c) and 2(d)). Given that CD40L is primarily expressed on antigen-stimulated CD4^+^ T cells [13], we reasoned that CD40 signaling might be involved in the regulation of B cell traffic during an immune response through promoting CXCR5 expression in B cells. In addition, other non-B cells also contributed to the optimal expression of CXCR5 in B cells through cell-cell contact and cytokine secretion (Figures 2(c) and 2(d)). Taken together, these results demonstrated that CD40 signaling promoted CXCR5 expression in a B cell-intrinsic manner, while non-B cells also contributed to the optimal expression of CXCR5 in B cells.

### 3.3. CD40-Mediated Activation of Noncanonical NF-*κ*B Signalling Contributed to CXCR5 Expression in B Cells

**Figure 3:**
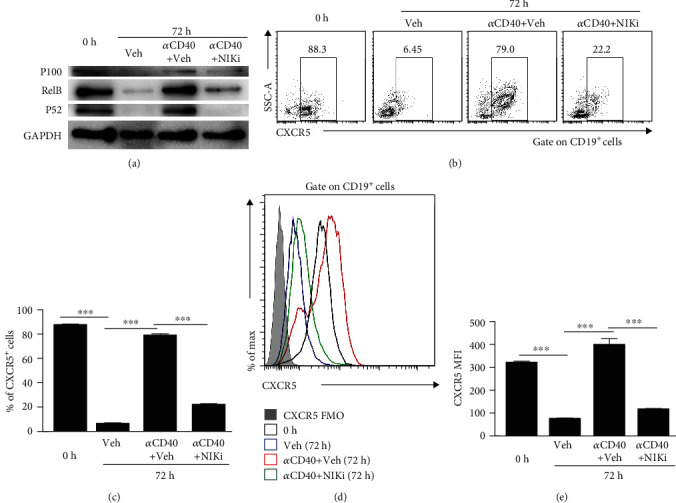
Noncanonical NF-*κ*B pathway was involved in CD40-mediated CXCR5 expression in B cells. Splenocytes were stimulated with anti-mouse CD40 antibody for 72 hours in the presence or absence of NIK inhibitor. (a) A representative immunoblot analysis showing P100, P52, and RelB in B cells. GAPDH was used as loading control (representing three independent experiments, *n* = 3); (b, c) Representative flow cytometry data plots (b) and statistics (c) showed the frequencies of CXCR5^+^ B cells (representing three independent experiments, *n* = 3); (d, e) Representative flow cytometry data plots (d) and statistics (e) showed the MFI of CXCR5 on B cells (representing three independent experiments, *n* = 3). Living cells were gated according to FSC and SSC parameters. All flow cytometry results were analysed and plotted using FMO. ^∗∗∗^*P* < 0.001 (ANOVA test).

Given that CD40 signaling in B cells induced the activation of the noncanonical NF-*κ*B pathway, we wondered whether noncanonical NF-*κ*B signalling was involved in CD40-mediated induction of CXCR5 expression in B cells. Notably, the expressions of RelB and P52, key regulators of the noncanonical NF-*κ*B pathway, were strongly increased in B cells treated with anti-CD40 agonistic antibody (Figure 3(a)). While inhibition of NF-*κ*B-inducing kinase (NIK), a central component of the noncanonical NF-*κ*B pathway, considerably impaired CD40-mediated CXCR5 expression in B cells, reflected by decreases in the percentages of CXCR5^+^ B cells (Figures 3(b) and 3(c)) and the MFI of CXCR5 in B cells (Figures 3(d) and 3(e)), which was associated with reduced levels of RelB and P52 (Figure 3(a)). In conclusion, our data indicated that CD40 signaling promoted the expression of CXCR5 in B cells at least partially through noncanonical NF-*κ*B pathway activation.

## 4. Conclusions

CXCR5-mediated signals have a pivotal role in regulating B cell trafficking, which is important for orchestrating the functions of B cells including supporting follicular helper T-cell generation and producing antibodies. We showed that CD40 signaling promoted the expression of CXCR5 in a B cell-intrinsic manner in part through noncanonical NF-*κ*B pathway activation, while other non-B cells were involved in the optimal expression of CXCR5 in B cells. Our findings suggest that CD40-mediated induction of CXCR5 expression may contribute to orchestrating B cell function through controlling B cell trafficking.

## Data Availability

All data are fully available without restriction.

## Supplementary Materials

Supplementary Materials Supporting Information Figure 1: BAFF promotes CXCR5 expression in B cells. Supporting Information Figure 2: CD40 signal promotes CXCR5 expression in B cells.
