# Supplementary material for: CD40 Signaling Promotes CXCR5 Expression in B Cells via Noncanonical NF-κB Pathway Activation
Source: J Immunol Res. 2020 Jul 31;2020:1859260. doi: 10.1155/2020/1859260 (PMC7415097; doi:10.1155/2020/1859260)
Supplement: Supplementary Materials — Supporting Information Figure 1: BAFF promotes CXCR5 expression in B cells. Supporting Information Figure 2: CD40 signal promotes CXCR5 expression in B cells. [file 1859260.f1.docx]

**Supporting Information Figures and Figure Legends**


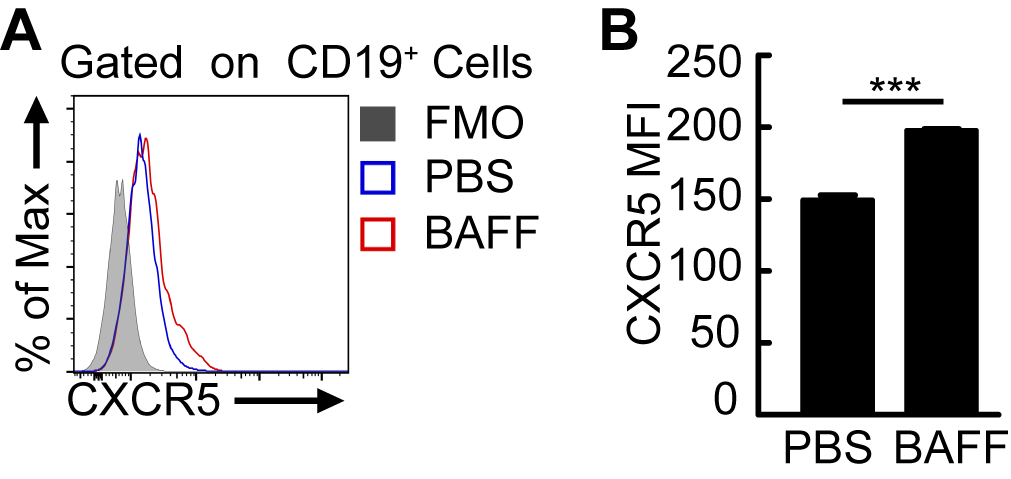


**Supporting Information Figure 1 BAFF promotes CXCR5 expression in B cells**

(A) Representative flow cytometry data plots showed the MFI of CXCR5 on B cells *in vitro* stimulated with or without BAFF for 72 hours (representing three independent experiments, n=3). (B) Flow cytometry data statistics showed the MFI of CXCR5 on B cells. Data were representative of three independent experiments and presented as mean + s.d. (n=3). Live cells were gated according to FSC and SSC parameters. Flow cytometry results were analysed and plotted using FMO. ***P＜0.001. (Student’s *t*-test).


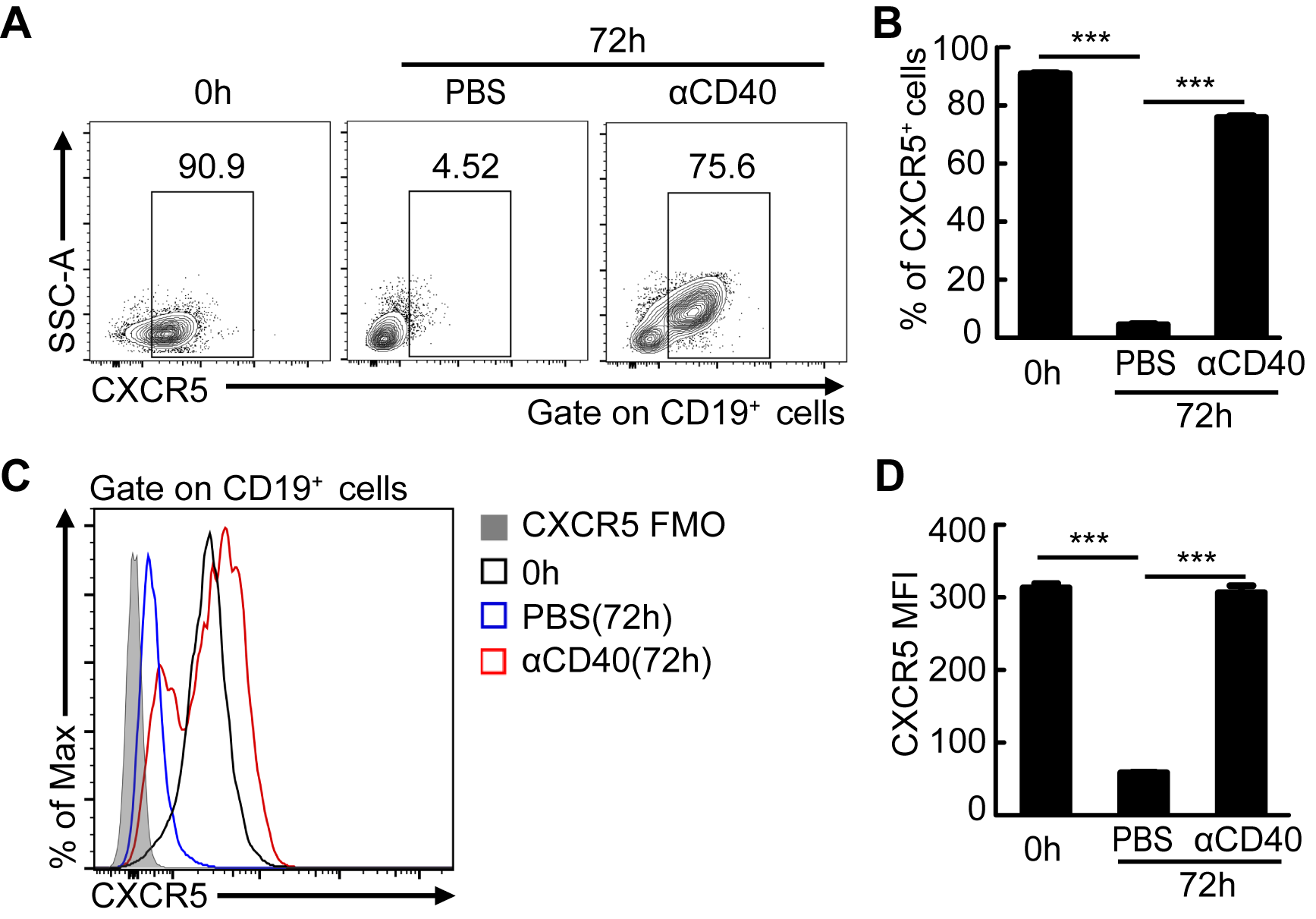


**Supporting Information Figure 2 CD40 signal promotes CXCR5 expression in B cells**

(A-B) Representative flow cytometry data plots (A) and statistics (B) showed the frequencies of CXCR5^+^ B cells (representing three independent experiments, n=3); (C-D) Representative flow cytometry data plots (C) and statistics (D) showed the MFI of CXCR5 on B cells (representing three independent experiments, n=3). Live cells were gated according to FSC and SSC parameters. All flow cytometry results were analysed and plotted using FMO. ***P＜0.001 (ANOVA test).
